# Effect of Bimagrumab on body composition: a systematic review and meta-analysis

**DOI:** 10.1007/s40520-024-02825-4

**Published:** 2024-09-09

**Authors:** Mehmet Kanbay, Dimitrie Siriopol, Sidar Copur, Nuri Baris Hasbal, Mustafa Güldan, Kam Kalantar-Zadeh, Tania Garfias-Veitl, Stephan von Haehling

**Affiliations:** 1 Division of Nephrology, Department of Medicine, Koc University School of Medicine, Istanbul, Turkey; 2 Department of Nephrology, “Saint John the New” County Hospital, Suceava, Romania; 3 ”Stefan Cel Mare” University, Suceava, Romania; 4 Department of Medicine, Koc University School of Medicine, Istanbul, Turkey; 5 Division of Nephrology and Hypertension, Department of Medicine, UCLA Medical Center, Harbor, Torrance, CA USA; 6 UCLA David Geffen School of Medicine, Los Angeles, CA USA; 7 The Lundquist Institute for Biomedical Innovation at Harbor, UCLA Medical Center, Torrance, CA USA; 8 Tibor Rubin VA Medical Center, Long Beach VA Healthcare System, Long Beach, CA USA; 9 Department of Cardiology and Pneumology, University of Medical Center Göttingen, Robert-Koch-Str. 40, 37075 Göttingen, Germany

**Keywords:** Sarcopenia, Bimagrumab, Strength, Treatment modality, Thigh muscle volume, Physical performance, Fat-free body mass, Fat body mass

## Abstract

**Background:**

Sarcopenia, a condition marked by progressive muscle mass and function decline, presents significant challenges in aging populations and those with chronic illnesses. Current standard treatments such as dietary interventions and exercise programs are often unsustainable. There is increasing interest in pharmacological interventions like bimagrumab, a monoclonal antibody that promotes muscle hypertrophy by inhibiting muscle atrophy ligands. Bimagrumab has shown effectiveness in various conditions, including sarcopenia.

**Aim:**

The primary objective of this meta-analysis is to evaluate the impact of bimagrumab treatment on both physical performance and body composition among patients diagnosed with sarcopenia.

**Materials and methods:**

This meta-analysis follows the Preferred Reporting Items for Systematic Reviews and Meta-Analyses (PRISMA) guidelines. We systematically searched PubMed, Ovid/Medline, Web of Science, and the Cochrane Library databases up to June 2024 using appropriate Medical Subject Headings (MeSH) terms and keywords related to bimagrumab and sarcopenia. Eligible studies were randomized controlled trials (RCTs) that assessed the effects of bimagrumab on physical performance (e.g., muscle strength, gait speed, six-minute walk distance) and body composition (e.g., muscle volume, fat-free body mass, fat body mass) in patients with sarcopenia. Data extraction was independently performed by two reviewers using a standardized form, with discrepancies resolved through discussion or consultation with a third reviewer.

**Results:**

From an initial search yielding 46 records, we screened titles, abstracts, and full texts to include seven RCTs in our meta-analysis. Bimagrumab treatment significantly increased thigh muscle volume (mean difference [MD] 5.29%, 95% confidence interval [CI] 4.08% to 6.50%, P < 0.001; moderate heterogeneity χ2 = 6.41, I2 = 38%, P = 0.17) and fat-free body mass (MD 1.90 kg, 95% CI 1.57 kg to 2.23 kg, P < 0.001; moderate heterogeneity χ2 = 8.60, I2 = 30%, P = 0.20), while decreasing fat body mass compared to placebo (MD − 4.55 kg, 95% CI − 5.08 kg to − 4.01 kg, P < 0.001; substantial heterogeneity χ2 = 27.44, I2 = 89%, P < 0.001). However, no significant improvement was observed in muscle strength or physical performance measures such as gait speed and six-minute walk distance with bimagrumab treatment, except among participants with slower baseline walking speeds or distances.

**Discussion and conclusion:**

This meta-analysis provides valuable insights into the effects of bimagrumab on sarcopenic patients, highlighting its significant improvements in body composition parameters but limited impact on functional outcomes. The observed heterogeneity in outcomes across studies underscores the need for cautious interpretation, considering variations in study populations, treatment durations, and outcome assessments. While bimagrumab shows promise as a safe pharmacological intervention for enhancing muscle mass and reducing fat mass in sarcopenia, its minimal effects on muscle strength and broader physical performance suggest potential limitations in translating body composition improvements into functional gains. Further research is needed to clarify its long-term efficacy, optimal dosing regimens, and potential benefits for specific subgroups of sarcopenic patients.

**Supplementary Information:**

The online version contains supplementary material available at 10.1007/s40520-024-02825-4.

## Introduction

The European Working Group on Sarcopenia in Older People-2 defines sarcopenia as reduced muscle mass and/or muscle strength as assessed via grip strength or gait speed by [1]. Whilst it affects 5–16% of elderly people as a whole, it is more commonly encountered in younger patients with significant medical conditions such as malignancies, chronic kidney disease, liver cirrhosis, heart failure or cerebrovascular disease [2]. Sarcopenia has been associated with poor quality of life, higher rates of morbidity and mortality, higher rates of hospitalizations, and higher risk of various medical comorbidities including osteoporosis, cognitive impairment, metabolic syndrome, hypertension and depression [2]. Currently, the available management options for sarcopenia include physical exercise programs such as aerobic exercise, resistance training, high-intensity interval training and whole-body vibration therapy as well as dietary modifications including high-protein nutritional supplements, supplementation with vitamin D and anti-oxidant agents [3]. Nevertheless, such physical therapy modalities may not be suitable for a large proportion of patients either due to reduced physical activity capacity or their general medical status. Therefore, with several clinical studies yielding neutral or disappointing results, there is growing interest in developing novel pharmacotherapeutic approaches for the management of sarcopenia [4, 5].

Bimagrumab is a monoclonal antibody that targets both the activin type 2A and B, which are mediators of several TGF-beta family proteins such as activins and myostatin. Blockage of these protein ligands is responsible for muscle atrophy. Activation of Act2RA and Act2RB supports differentiation of human myoblasts [6]. By doing so, it can promote muscle hypertrophy in animals [6] and humans [7] which has an impact on various conditions, including sarcopenia, body myositis, casting-induced disuse atrophy, recovery after hip fractures and chronic obstructive pulmonary disease [8–12]. Its effects are thought to result from the attenuation of negative regulators of muscle mass, such as myostatin [6, 7]. Myostatin, activin A, activin B, and growth and differentiation factor 11 are negative regulators that inhibit skeletal muscle mass through activin type 2 receptors [11]. It has been shown that both humans and animals with genetic mutations that reduce or eliminate myostatin have increased muscle mass, but are otherwise healthy [13, 14].

In the present systematic review and meta-analyzes we sought to evaluate, the efficacy of variable dosing regimens of bimagrumab in adult populations on the course of sarcopenia. Both age-related and medical condition-associated forms of sarcopenia were included in assessing measures of physical activity or muscle strength or techniques measuring muscle mass.

## Materials and methods

The PRISMA (Preferred Reporting Items for Systematic Reviews and Meta-Analyses) standards were followed for conducting this meta-analysis [15]. There were no deviations from the search strategy and pre-established methods by authors, emphasizing a full transparency.

### Data source and search strategy

**Fig. 1:**
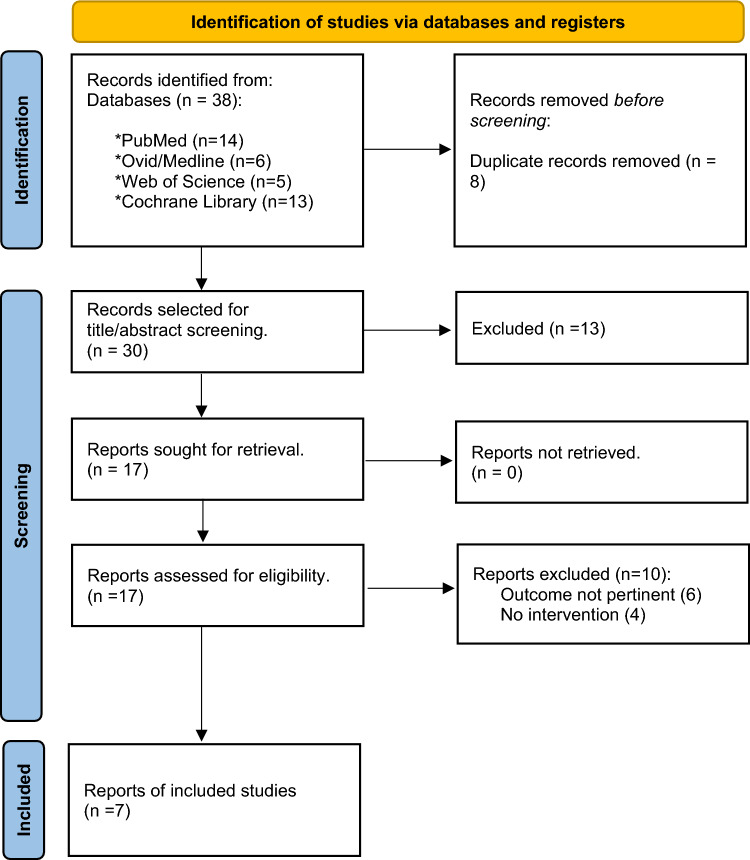
Flow diagram of the study selection process

PubMed, Ovid/Medline, Web of Science and Cochrane Library databases were used with the search strategies outlined in Fig. 1. The search was limited to studies published between 1960 through June 2024. Studies published in a peer-reviewed journal in English were included. Additionally, the selected keywords and steps during the search in each database are in detail in Supplementary Table 1. The search criteria were designed and performed by two authors (M.K., S.C.).

### Inclusion and exclusion criteria

We included randomized controlled trials (RCTs) that focused on patients diagnosed with sarcopenia and investigated the effects of bimagrumab administration. Eligible studies reported outcomes related to either body composition, such as thigh muscle volume, fat-free body mass, or fat body mass, or physical performance measures like voluntary knee extension strength, hand grip strength, gait speed, and six-minute walk distance. Studies had to be published in peer-reviewed journals and available in English to ensure comprehensive coverage of relevant literature and facilitate clear synthesis of findings.

We excluded non-randomized studies, including observational studies, retrospective or prospective cohort studies, case reports, case series, reviews, and meta-analyses, as they do not provide the rigorous evidence necessary for this systematic review. Studies involving patients who did not meet the diagnostic criteria for sarcopenia or included individuals under the age of 18 were also excluded. Additionally, studies that did not administer bimagrumab as part of their intervention or did not report outcomes related to body composition or physical performance were not considered. Non-English language publications and duplicate reports of the same study were also excluded to maintain clarity and consistency in the review process and to focus on the most relevant and robust evidence available.

Two investigators (M.K. and S.C.) independently screened abstracts and titles of the studies that were reached through the search platforms mentioned above. Bibliographies of the reviews and studies were additionally screened for relevant publications. Discrepancies were resolved by consensus third author D.S.. The selected studies were further investigated by two investigators (M.K. and S.C.) in full text, according to the criteria specified, and were reviewed by M.K. Further, references listed on selected studies and reviews were assessed manually for additional relevant studies. After the preliminary selection, the full texts of the selected studies were evaluated by authors independently. Details of the study selection procedures are depicted in Fig. 1.

Systematic reviews conducted exclusively in English, like in our case, offer several compelling advantages over reviews that include multiple languages. Firstly, focusing on English-only literature ensures a comprehensive coverage of studies from leading academic journals and databases where English is predominantly used. This approach minimizes the risk of missing key research findings that might be less accessible or indexed differently in other languages. Secondly, standardizing the language of publication enhances the consistency and clarity of the review process, facilitating a more coherent synthesis of evidence. This clarity not only improves the accessibility of findings to a wider audience but also enhances the reliability and reproducibility of the review's conclusions. Indeed, limiting systematic reviews to English-language publications has been already shown to exert minimal influence on the effect estimates and overall conclusions drawn from them [16].

Two authors (M.K. and S.C.) were responsible for collecting data from the studies. They extracted various information related to the studies, including their characteristics such as the year of performing and publishing the study, first author, and study design, as well as population characteristics such as age, sex, body mass index (BMI) and HbA1c levels. The authors collected information on thigh muscle volume, fat-free body mass and fat body mass, voluntary knee extension strength, hand grip strength, gait speed, and six-minute walk distance. The collected information is presented in Table 1 and Table 2.

**Table 1:** Clinical characteristics of the included studies

Study	Study design	Study duration	Sarcopenia definition	Intervention	Outcome measures	Funding
Rooks et al. 2020 a [10]	Double-blind placebo-controlled RCT	24 weeks	Gait speeds greater than 4 m of 0.3 m/s or more to less than 0.8 m/s, and appendicular skeletal muscle index (ASMI; kg/m2) values meeting cutoffs for non-Asian11 and Asian13 countries	Bimagrumab therapy versus Optimized standard point of care Dietary intervention (> 20 kcal/kg of body weight calories/day and > 0.8 g/kg of body weight/day protein) and vitamin D supplementation for all participants	-Total SPPB score -6MWD -Gait speed -Fat-free body mass and fat body mass assessed via DXA -Handgrip strength assessed via dynamometer -Self-reported falls -Patient-reported outcomes assessed via European Quality of Life Dimensions-5 levels	Novartis Institutes for BioMedical Research
Polkey et al. 2019 [11]	Double-blind placebo-controlled phase 2a RCT	24 weeks	BMI < 20 kg/m2 ASMI < 5.45 kg/m2 for females and < 7.45 kg/m2 for males	Bimagrumab therapy versus Optimized standard point of care Dietary intervention (> 20 kcal/kg of body weight calories/day and > 0.6 g/kg of body weight/day protein) for all participants	-Thigh muscle volume assessed via MRI -6MWD -Total and appendicular fat-free body mass and fat mass assessed via DXA -Handgrip strength assessed via dynamometer -Stair climbing time -Bilateral one-repetition maximum leg press	Novartis Institutes for BioMedical Research, National Institute for Health Research Respiratory Biomedical Research Unit at the Royal Brompton and Harefield National Health Service Foundation Trust and Imperial College, London, UK
Rooks et al. 2017 b [7]	Double-blind placebo-controlled parallel-arm RCT	24 weeks	Not reported	Bimagrumab therapy versus Optimized standard point of care Dietary intervention (> 20 kcal/kg of body weight calories/day and > 0.6 g/kg of body weight/day protein) for all participants	-6MWD -Gait speed -Hand Grip strength -LBM -TMV, IMAT and SCAT assessed via MRI	Novartis Institutes for BioMedical Research
Rooks et al 2017 a [8]	double-blind, placebo-controlled trial	12 weeks	N/A	Bimagrumab therapy versus Placebo Using a full-length cast to one of the lower extremities for 2 weeks period, to eventually induce disuse atrophy before randomization	Changes in -Thigh muscle volume (TMV) -Inter-muscular adipose tissue (IMAT) -Subcutaneous adipose tissue (SCAT) of the thigh, -Maximum voluntary knee extension strength	Novartis Institutes for BioMedical Research
Heymsfield et al. 2021 [19]	Phase 2 RCT on patients with type II diabetes mellitus	48 weeks	N/A	Bimagrumab therapy versus Optimized standard point of care Dietary intervention (Caloric restriction) and physical activity for all participants	-Body fat mass assessed via DXA -Waist circumference -Hepatic fat fraction assessed via MRI -Subcutaneous and abdominal fat mass assessed via MRI -Handgrip strength by dynamometry	Novartis Institutes for BioMedical Research
Hofbauer et al. 2021 [12]	Multicenter double-blind placebo-controlled RCT	24 weeks	N/A	Bimagrumab therapy at various doses versus Optimized standard point of care	-Fat-free body mass assessed via DXA -SPPB score and habitual gait speed -Self-reported falls	Novartis Institutes for BioMedical Research
Rooks et al. 2020 b [18]	Double-blind placebo-controlled RCT	20 weeks and 12 weeks	N/A	Bimagrumab therapy versus Optimized standard point of care	-BMI -TMV -Total LBM -Total FBM -Bilateral leg press	Novartis Institutes for BioMedical Research, Cambridge, MA, USA, and Basel, Switzerland

*RCT* randomized control trial, *BMI *body mass index, *6MWD* 6-min walking distance, *LBM* lean body mass (Fat-free body mass), *TMV* thigh muscle volume, *FEV* forced expiratory volume, *FVC* forced vital capacity, *ALM* appendicular lean mass, *MRI* magnetic resonance imaging, *DXA* dual X-ray absorptiometry, *SPPB *short physical performance battery, *IMAT *intermuscular adipose tissue, *SCAT* subcutaneous adipose tissue, *FBM* fat body mass, *n*-number, *N/A* not applicable

**Table 2:** Clinical characteristics of the included studies in terms of participants’ characteristics and outcomes

Study	Characteristics of the Bimagrumab group	Characteristics control group	Endpoints
Rooks et al. 2020 a [10]	Bimagrumab 700 mg (n = 113) -Mean age: 79.5 years -Gender: 41.6% Male -Mean BMI: 24 kg/m2 -6MWD: 294.3 -Total SPPB score: 7.1	Placebo n = 67 -Mean age: 78.3 years -Gender: 35.8% Male -Mean BMI: 23.6 kg/m2 -6MWD: 312.4 -Total SPPB score: 7.1	-No statistically significant difference has been recorded in terms of total SPPB score, 6MWD or gait speed -Bimagrumab therapy increases fat-free body mass compared to optimized standard care (p-value < 0.001)
Polkey et al. 2019 [11]	- Bimagrumab 30 mg/kg (n = 33) -Mean age: 64.5 years -Gender: 51% Male -Mean BMI: 19.5 kg/m2 -FEV1/FVC: 36.1% -LBM: 35.5 kg -6MWD: 361 m	Placebo n = 34 -Mean age: 63.1 years -Gender: 47% Male -Mean BMI: 19.1 kg/m2 -FEV1/FVC: 38.9% -LBM: 33.6 kg -6MWD: 372 m	-Bimagrumab therapy improves TMV (p-value < 0.001) and LBM (p-value < 0.001) and significantly declines intermuscular or subcutaneous or appendicular adipose tissue assessed by MRI -Bimagrumab therapy has no significant effect on muscle strength mobility or respiratory parameters
Rooks et al. 2017X [7]	- Bimagrumab 30 mg/kg (n = 19) -Mean age: 71.6 years -Gender: 68% Male -Mean BMI: 24.9 kg/m2 -6MWD: 294 m -Gait speed: 0.78 m/s -LBM: 38.2 kg	Placebo n = 21 -Mean age: 72.4 years -Gender: 38% Male -Mean BMI: 26.2 kg/m2 -6MWD: 307.7 m -Gait speed: 0.82 m/s -LBM: 36.9	-Bimagrumab therapy improves TMV (p-value = 0.002), LBM (p-value = 0.003), ALM (p-value < 0.001) and intermuscular (p-value < 0.05) or total body fat mass (p-value < 0.001) -Bimagrumab therapy improves 6MWD in participants with short baseline walk distance and gait speed in patients with slower baseline walking speed
Heymsfield et al. 2021 [19]	- Bimagrumab 10 mg/kg (up to a maximum 1200 mg) (n = 37) -Mean age: 60.7 years -Gender: 38% Male -Mean BMI: 32.7 kg/m2 -Mean HbA1c: 7.99%	Placebo n = 38 -Mean age: 60.2 years -Gender: 68% Male -Mean BMI: 33.1 kg/m2 Mean HbA1c: 7.66%	-Bimagrumab therapy reduces body fat mass (p-value < 0.001), waist circumference (p-value < 0.001), HbA1c (p-value = 0.005) and increases fat-free body mass (p-value < 0.001) -Bimagrumab therapy has been linked to significant decline in hepatic fat fraction (p-value = 0.01), abdominal visceral adipose tissue (p-value = 0.01) and non-significant decline at subcutaneous adipose tissue (p-value = 0.07)
Hofbauer et al. 2021 [12]	i) Bimagrumab 70 mg (n = 34): -Mean age: 76.1 years -Gender: 38% Male -Mean BMI: 24.8 kg/m2 -Mean TLB: 36.2 kg ii) Bimagrumab 210 mg (n = 69): -Mean age: 74.8 years -Gender: 30% Male -Mean BMI: 24.7 kg/m2 -Mean TLB: 36.1 kg iii) Bimagrumab 700 mg (= 75): -Mean age: 76.1 years -Gender: 28% Male -Mean BMI: 24.2 kg/m2 -Mean TLB: 34.4 kg	Placebo n = 72 -Mean age: 76.4 years -Gender: 26% Male -Mean BMI: 24.4 kg/m2 -Mean TLB: 34.9 kg	-Bimagrumab therapy at 210 mg and 700 mg leads to statistically significant and dose-dependent improvement in fat-free body mass (p-value < 0.001) -Bimagrumab therapy leads to improvement in gait speed and SPPB score but none of those reaches statistical significance
Rooks et al 2020 b [18]	i) Bimagrumab 3 mg/kg older adult (n = 6): -Mean age: 74.5 years -Gender: 0% Male -Mean BMI: 26.7 kg/m2 ii) Bimagrumab 30 mg/kg older adult (n = 6): -Mean age: 73 years -Gender: 33.3% Male -Mean BMI: 28.6 kg/m2 iii) Bimagrumab 30 mg/kg obese adult (n = 6): -Mean age: 40.2 years -Gender: 83.3% Male -Mean BMI: 33 kg/m2	i) or ii)Placebo older adult (n = 4): -Mean age: 76.8 years -Gender: 75% Male -Mean BMI: 23.3 kg/m2 iii) Placebo obese adult group (n = 2): -Mean age: 41 years -Gender: 0% Male -Mean BMI: 38 kg/m2	-Bimagrumab therapy leads to improvement in TMV and LBM -Bimagrumab therapy is not associated with any significant adverse effect
Rooks et al. 2017 a [8]	Bimagrumab 30 mg/kg (n = 15): -Mean age: 23.5 years -Gender: 100% Male -Mean BMI: 25.3 kg/m2 -TMV: 5237.9	Placebo group (n = 9): -Mean age: 25.1 years -Gender: 100% Male -Mean BMI: 25.3 kg/m2 -TMV: 5010.4	-Bimagrumab therapy leads to statistically significant improvement in TMV and a decline in IMAT -Bimagrumab therapy has not been associated with any considerable adverse effect

*RCT* randomized control trial, *BMI* body mass index, *6MWD* 6-min walking distance, *LBM* lean body mass (Fat-free body mass), *TMV* thigh muscle volume, *FEV* forced expiratory volume, *FVC *forced vital capacity, *ALM* appendicular lean mass, *MRI* magnetic resonance imaging, *DXA *dual X-ray absorptiometry, *SPPB *short physical performance battery, *IMAT* intermuscular adipose tissue, *SCAT* subcutaneous adipose tissue, *FBM* fat body mass, *n* number, *N/A *not applicable

### Risk of *bias* assessment

Risk of bias within included studies was systematically assessed using the Cochrane Collaboration's tool, evaluating random sequence generation, allocation concealment, blinding of participants and personnel, blinding of outcome assessment, incomplete outcome data, selective reporting, and other potential sources of bias (Supplementary Table 2). Any discrepancies in data extraction or risk of bias assessments were resolved through consensus or consultation with a third reviewer.

### Study objective

Our investigation must include studies in which bimagrumab was administered to individuals with sarcopenia along with assessments of either physical performance or body composition.

### Data analysis

We investigated the effect of Bimagrumab on continuous outcomes using a two-tailed variance analysis in samples with known arithmetic means and standard deviations. Generic inverse variance based on calculating absolute differences of mean changes between the experimental and control groups and standard deviations for each comparison within each study were used. We converted the standard error and 95% confidence interval (CI) to standard deviation by using a standard formula [17].

If data were reported at more than one-time point during the study, we used the end-of-treatment data. If a study had more than two intervention arms, the control group sample size was split by the number of subgroup comparisons for that study. The treatment effect was significant if p < 0.05. We assessed for heterogeneity in treatment estimates using the Cochrane Q test and the χ^2^ statistic (with substantial heterogeneity defined as values > 50%). We conducted a sensitivity analysis to assess the contribution of each study to the pooled treatment effect by excluding each study one at a time and recalculating the pooled treatment effect for the remaining studies (leave-one-out meta-analysis).

Analyses were performed with the Review Manager (Version 5.3, The Cochrane Collaboration 2012).

## Results

### Selection and description of studies

Our analysis included seven RCTs. The total number of patients included was 660 (minimum 24 [8, 18] and maximum 250 [12] patients) with a follow-up period between 12 [8] and 48 weeks [19]. Except for one study [8] which included only men, all other studies assessed both sexes. Three studies were performed in the USA [7, 8, 18] and 4 were multicentric [10–12, 19].

Rooks, Laurent et al. (Rooks 2017a) included young healthy participants [8]; the same group later evaluated individuals with older age [7, 10, 18] (Rooks 2017b, Rooks 2020a, Rooks 2020b) or obesity (Rooks 2020b) [18] in three different studies. Polkey et al. assessed the effect of bimagrumab in chronic obstructive pulmonary disease [11], while Heymsfield et al. included patients with obesity or diabetes mellitus [19]. The most recent study evaluated older patients who had undergone internal fixation or hemiarthroplasty for a proximal femoral fracture [12].

The doses of bimagrumab were different between studies. A single dose of 30 mg/kg bimagrumab was used in two studies [8, 18]. Additionally, in one of these studies, a single dose of 3 mg/kg was used (in the older subgroup of patients [10]). Two studies used two doses of 30 mg/kg bimagrumab (at baseline and 8 weeks) [7, 11]. The rest of the studies administered bimagrumab at 4 weeks – 700 mg [10], 10 mg/kg (with a maximum dose of 1200 mg) [19] and 70 mg, 210 mg or 700 mg [12].

### Body composition

**Fig. 2:**
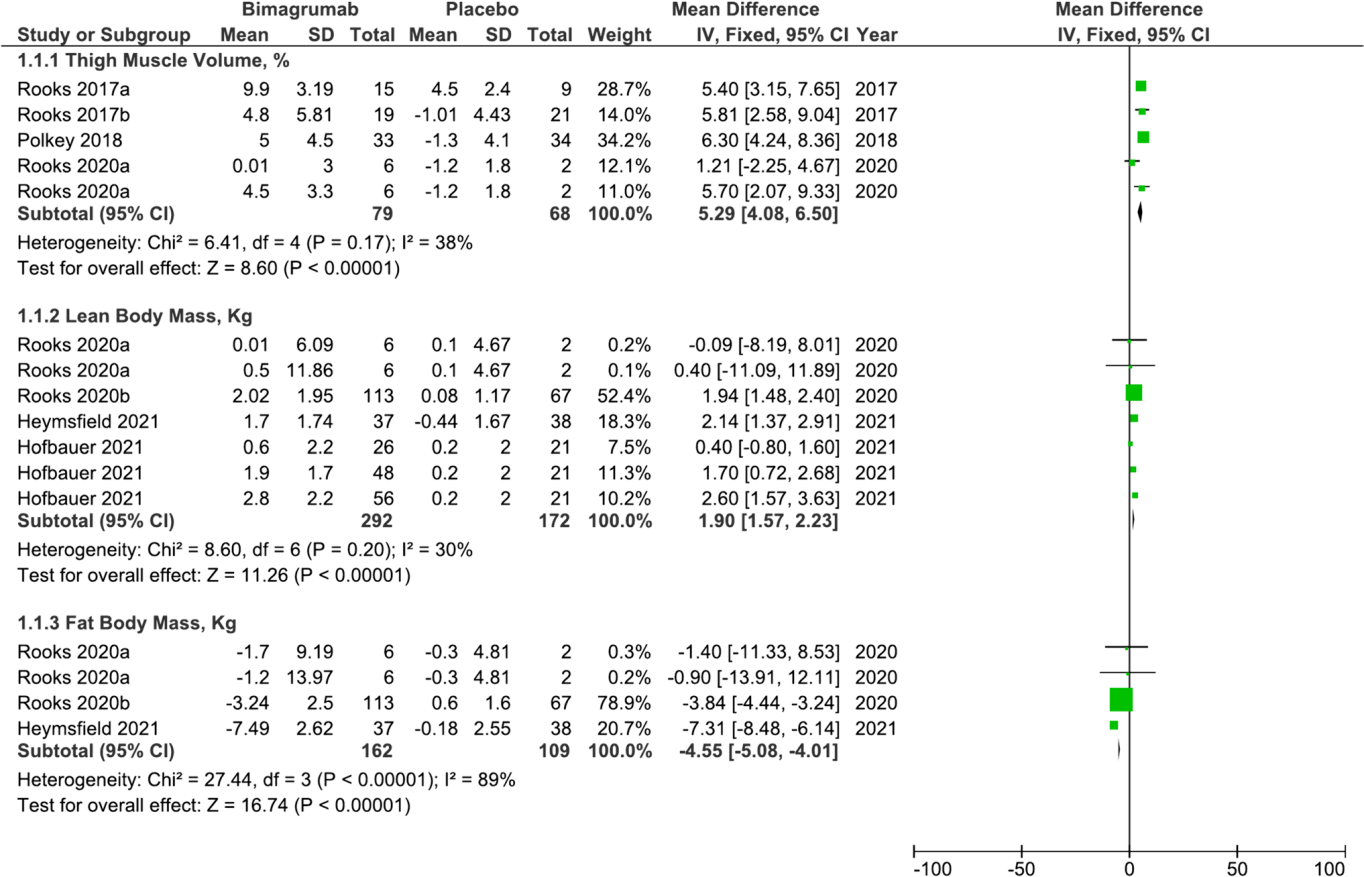
Forest plot of the included studies for the effect of bimagrumab on thigh muscle volume, fat-free body mass and fat body mass

4 studies analyzed the effect of bimagrumab on thigh muscle volume (TMV) [7, 8, 10, 11]. Overall, there was a significant increase in TMV levels with bimagrumab treatment (Mean Difference (MD) 5.29%, 95% Confidence Interval (CI) 4.08% to 6.50%, P < 0.001; heterogeneity χ^2^ = 6.41, I^2^ = 38%, P = 0.17) (Fig. 2, 1.1.1). The effect of bimagrumab on fat-free body mass (LBM) was assessed in 5 studies [8, 10, 12, 18, 19]. As shown in Fig. 2, 1.1.2, bimagrumab treatment significantly increased fat-free body mass (MD 1.90 kg, 95% CI 1.57 kg to 2.23 kg, P < 0.001; heterogeneity χ^2^ = 8.60, I^2^ = 30%, P = 0.20). As compared with placebo, bimagrumab was also effective in reducing fat body mass (MD − 4.55 kg, 95% CI − 5.08 kg to − 4.01 kg, P < 0.001; heterogeneity χ^2^ = 27.44, I^2^ = 89%, P < 0.001) (Fig. 2, 1.1.3). [8, 10, 12, 18, 19] Although not included in the meta-analysis because of unit incompatibility of the results, Rooks, Laurent et al. [8] also identified an increase in fat-free body mass and a reduction in fat body mass with bimagrumab treatment (Supplementary Table 3).

### Physical performance

Voluntary knee extension strength was assessed in 2 studies [8, 10], and no change in muscle strength was detected in the bimagrumab-treated groups. The effect of treatment on hand grip strength was mixed. Although a minimally, but significant, increase was noted by Rooks et al. [8] at different time points during the study period, no changes were seen in the other two other studies [10, 19]. Similarly, there was no significant difference between bimagrumab and placebo on gait speed [7, 8, 10, 12] or the six-minute walk distance [8, 10], although a sub-analysis of one of the studies suggested that participants with slower walking speed (< 0.8 m/s) or lower 6-min walking distance (< 300 m) at baseline who received bimagrumab consistently increased their gait speed (0.15 m/s) or walking distance (118 m) more than those on placebo [7].

### Sensitivity analysis and evaluation of publication *bias*

The leave-one-out type of analysis was used to assess the influence of each individual study on the overall pooled effect estimate, but also on the heterogeneity of these results. Using this approach, we noticed that most of the heterogeneity observed for the Fat Body Mass analysis was due to the study by Heymsfield et al., suggesting an increased effect of bimagrumab in reducing fat mass in obese and diabetic patients (although this is the study that used the highest doses of bimagrumab, it didn’t influence the heterogeneity in the Lean Body Mass analysis).

**Fig. 3:**
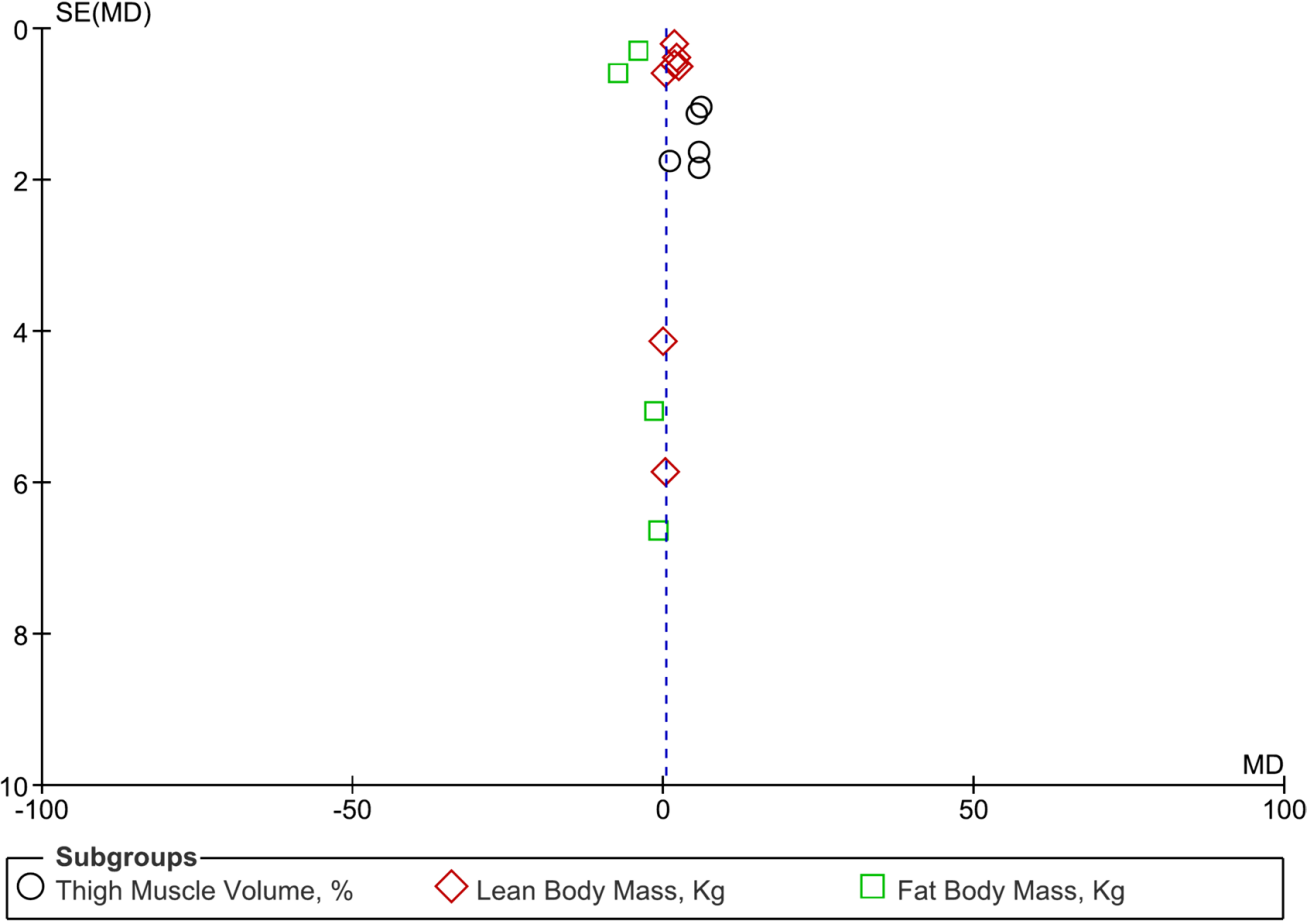
Funnel plot of the mean differences in thigh muscle volume, lean body mass and fat body mass versus standard errors of the mean differences The x-axis is in % (for thigh muscle volume) or Kg (for lean body mass and fat body mass)

With the limitation of a low number of studies included, the funnel plot (Fig. 3) shows a rather symmetrical plot for each of the three outcomes, which makes reporting bias improbable using the type of assessment.

## Discussion

Sarcopenia, defined by the presence of low muscle strength, muscle quantity or quality and low physical performance leading to increased risk of adverse events such as falls, fractures and physical disability, has a varying prevalence ranging between 2.5% to 35% depending on the study population with higher rates in elderly populations and depending on the method of investigation and diagnostic criteria utilized [1, 20–22]. Although there are considerable variations in the diagnostic criteria in different guidelines, current methods for the evaluation of sarcopenia include bio-impedance analysis, dual-energy X-ray absorptiometry, handgrip strength, walking speed and imaging modalities such as computed tomography or magnetic resonance imaging [23]. Whilst resistance and strength training comprise the backbone of non-pharmacological treatment modalities, there is currently no pharmacotherapeutic approach approved by the United States Food and Drug Administration (FDA) for use in the management of sarcopenia. In this meta-analysis, we aimed to investigate the efficacy of bimagrumab in the management of sarcopenia in terms of alterations in muscle mass and muscle strength. We have shown that bimagrumab therapy leads to statistically significant improvements in fat-free body mass and TMV and a decline in body fat mass, however, no clinically relevant improvement has been recorded in muscle strength assessed via gait speed, six-minute walking distance or hand-grip strength. Such lack of correlation between fat-free body mass or TMV and muscle strength may be attributable to various factors including lack of neural adaptation including recruitment of motor units and de-activation of antagonist muscles, lack of resistance training and relatively short duration of follow-up in clinical trials for such a functional outcome to develop. There is a clear need for future large-scale clinical and pre-clinical studies investigating whether such discordance is related to those confounding factors.

Anabolic agents, frequently utilized in the management of sarcopenia, often yield augmented body mass in affected individuals by promoting muscle protein synthesis. However, their efficacy in enhancing muscle function remains variable and multifactorial. Several factors may contribute to this discordance. Firstly, anabolic agents may selectively target specific muscle fiber types, potentially neglecting those crucial for functional improvements. Secondly, age-related alterations in muscle composition, such as increased intramuscular fat and fibrosis, may impede the translation of increased mass into enhanced function [24]. Additionally, concomitant physical rehabilitation modalities are also effective to gain sufficient amount of strength beyond sole medical treatment [25]. Moreover, individual variability in treatment response, influenced by genetic, hormonal, and behavioral factors, can further confound the relationship between increased mass and improved function [26]. Lastly, inadequate dosages or durations of treatment, treatment compliance, may limit the therapeutic potential of anabolic agents in sarcopenic patients [27]. Understanding these intricacies is paramount in optimizing treatment strategies for sarcopenia, emphasizing the need for comprehensive approaches targeting both mass and function.

**Fig. 4:**
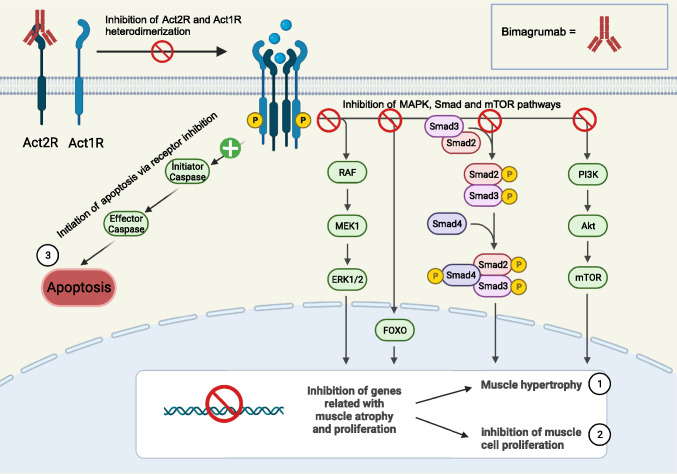
Cellular Signal Targets and Metabolic Effects of anti-Activin Type 2 Receptor Antibody Bimagrumab

Even though the exact underlying physiological mechanisms of sarcopenia are largely unknown, the activin/myostatin pathway appears to have a central role in the regulation of muscular growth and atrophy. The activin receptor pathway has a critical role in hyperplasia, hypertrophy and atrophy of skeletal muscle cells and is under the influence of various signals including therapeutic interventions. The binding of various ligands to activin type II receptors leading to heterodimerization with activin type I receptors activates the signalling pathway in which mitogen-activated protein kinases (MAPK) activation, suppression of mothers against decapentaplegic (Smad) and forkhead box transcription factors (FoxO) activation and phosphatidylinositol 3-kinase (PI3K)/protein kinase B (AKT)/mammalian target of rapamycin (mTOR) pathway inhibition occur [28]. The result is the inhibition of skeletal muscle cell proliferation and hypertrophy via the inhibition of genes involved in myogenesis and induction of apoptosis causing muscular atrophy [28]. Three major mechanisms have been proposed and investigated in pre-clinical and clinical studies including the use of anti-ligand, primarily against myostatin such as domagrozumab [29–31], the use of soluble activin type IIB receptor blockers, namely ACE-031 [32], and use of receptor antagonists such as bimagrumab (Fig. 4).

Another important therapeutic aspect of bimagrumab is patients with peripheral insulin resistance and obesity. A phase II RCT involving 75 participants with type II diabetes mellitus (HbA1c between 6.5–10%) and body-mass index of 28 to 40 kg/m2 has demonstrated statistically significant beneficial effects on fat-free body mass (+ 3.6% vs. − 0.8%, p-value < 0.001), total body fat mass (− 20.5% vs. − 0.5%, p-value < 0.001), HbA1c (− 0.76 vs. 0.04, p-value = 0.005) and total body weight (− 6.5% vs. − 0.8%, p-value < 0.001) over forty-eight week clinical trial period [19]. Similar patterns of improvement in fat-free body mass and total body fat mass have been demonstrated in another clinical trial involving sixteen participants with a mean body-mass index of 29.3 kg/m2 and insulin resistance after receiving a single dose of bimagrumab therapy [33]. Also, another study evaluating the efficiency and safety of bimagrumab therapy on elderly participants with obesity has illustrated effectiveness and safety on 24 participants [18]. Even though the initial clinical results of bimagrumab therapy in the management of obesity appear promising, current literature is primarily limited due to the inclusion of a low number of participants and there is a clear need for future large-scale clinical trials. Moreover, two clinical trials are being conducted to further evaluate such potential clinical use (NCT05933499, NCT05616013).

The major limitations of this meta-analysis study include the heterogeneity of included studies in terms of the methods and criteria utilized for the diagnosis and staging of sarcopenia, the underlying aetiology of sarcopenia, the duration and dosage of bimagrumab therapy, and the basic demographic characteristics of the study populations including age and sex. Such variations limit the generalizability of the results of our meta-analysis. Nevertheless, our meta-analysis study is investigating the efficacy and adverse effect profile of bimagrumab therapy in the management of sarcopenia, which is a growing medical concern, especially in the elderly. However, there is a clear need for future large-scale standardized clinical studies investigating the efficacy and adverse effect profile of bimagrumab therapy in the treatment of sarcopenia.

## Conclusion

This meta-analysis study aimed to investigate the effects of bimagrumab, a monoclonal antibody, on muscle mass and strength in adult patients with sarcopenia. The standard treatments for improving skeletal muscle mass and strength in older patients, such as dietary protein intake and resistance exercise training, can be challenging to maintain, so there is growing interest in developing pharmacological treatments that can counter muscle atrophy and enhance functional recovery. Bimagrumab therapy has a positive effect on body composition but does not appear to improve physical performance in the evaluated patient population, although it may be beneficial for those with slower baseline walking speed or distance, according to subgroup analyses. It is safe for individuals with elderly age, obesity and type 2 diabetes mellitus in several studies, making it a suitable candidate for future therapy options.

## Supplementary Information

Below is the link to the electronic supplementary material.

Supplementary file1 (DOCX 16 KB)

Supplementary file2 (DOCX 15 KB)

Supplementary file3 (DOCX 16 KB)

## Data Availability

No datasets were generated or analysed during the current study.
