# Supplementary material for: Effect of Bimagrumab on body composition: a systematic review and meta-analysis
Source: Aging Clin Exp Res. 2024 Sep 9;36(1):185. doi: 10.1007/s40520-024-02825-4 (PMC11385021; doi:10.1007/s40520-024-02825-4)
Supplement: Supplementary file 1 — Supplementary file1 (DOCX 16 KB) [file 40520_2024_2825_MOESM1_ESM.docx]

**Supplementary Table 1**. Search strategy detailed in Ovid-Medline, PubMed, Web of Science, Cochrane Library

| **OVID-Medline** |
| --- |
| Ovid MEDLINE(R) and Epub Ahead of Print, In-Process, In-Data-Review & Other Non-Indexed Citations and Daily <1946 to June, 2024>  1 sarcopenia.mp. or Sarcopenia/ or Muscular Atrophy/ (n=27795)  2 Clinical Trial.mp. or Clinical Trial/ (n=785902)  3 Treatment Outcome/ or randomized control trial.mp. (n=1143237)  4 2 or 3 (n=1786155)  5 Activin Receptors, Type II/ or bimagrumab.mp. (n=1247)  6 Body Composition.mp. or Body Composition/ (n=68741)  7 Body mass.mp. or Body Weight/ (n=498995)  8 Muscle Strength.mp. or Muscle Strength/ (n=45160)  9 Muscle Strength/ or Hand Strength/ or Hand grip.mp. (n=45504)  10 Physical Performance.mp. or Physical Functional Performance/ (n=14652)  11 6 or 7 or 8 or 9 or 10 (n=597021)  12 1 and 4 and 5 (n=9)  13 11 and 12 (n=6)  14 limit 13 to english language (n=6) |
| **PubMed** |
| English((("Sarcopenia"[Mesh]) OR sarcopenia OR "Muscular Atrophy"[Mesh]) OR muscular atrophy AND bimagrumab OR "bimagrumab" [Supplementary Concept]) AND "Clinical Trial" [Publication Type]  (n=14) |
| **Web of Science** |
| TITLE-ABS-KEY ( "sarcopenia" OR "Muscular Atrophy" ) AND TITLE-ABS-KEY ( "bimagrumab" OR "type 2 Activin Receptors" ) AND TITLE-ABS-KEY ( "randomized control trial" OR "clinical trial" ) AND TITLE-ABS-KEY ( "Body Composition" OR "Body mass" OR "body weight" OR "Muscle Strength" OR "Hand Strength" OR "Hand grip" OR "Physical Performance" OR "Physical Functional Performance" ) (n=13) |
| **COCHRANE LIBRARY** |
| # 1 (sarcopenia or Muscular Atrophy):ti,ab,kw (Word variations have been searched) (n=2961)  # 2 bimagrumab or type 2 Activin Receptors (n=58)  # 3 Treatment Outcome or randomized control trial or clinical trial (n=1277155)  # 4 Body Composition or Body mass or body weight or Muscle Strength or Hand Strength or Hand grip or Physical Performance or Physical Functional Performance (n=168932)  #1 AND #2 AND #3 AND #4 (n=13) |
