# Supplementary material for: Effect of Bimagrumab on body composition: a systematic review and meta-analysis
Source: Aging Clin Exp Res. 2024 Sep 9;36(1):185. doi: 10.1007/s40520-024-02825-4 (PMC11385021; doi:10.1007/s40520-024-02825-4)
Supplement: Supplementary file 2 — Supplementary file2 (DOCX 15 KB) [file 40520_2024_2825_MOESM2_ESM.docx]

**Supplementary table 2.** Risk of Bias Assessment Across Studies Included

| **Study Author** | **Study type** | **Risk of Bias** | **Inconsistency** | **Indirectness** | **Imprecision** | **Publication Bias** | **Overall** |
| --- | --- | --- | --- | --- | --- | --- | --- |
| **Rooks et al. 2017 b** | RCT | No concern | No concern | No concern | No concern | No concern | ⊕⊕⊕⊕ |
| **Rooks et al.**  **2017 a** | RCT | No concern | No concern | No concern | No concern | No concern | ⊕⊕⊕⊕ |
| **Rooks et al. 2020 a** | RCT | No concern | No concern | No concern | No concern | No concern | ⊕⊕⊕⊕ |
| **Polkey et al. 2019** | RCT | No concern | No concern | No concern | No concern | No concern | ⊕⊕⊕⊕ |
| **Hofbauer et al. 2021** | RCT | No concern | No concern | No concern | No concern | No concern | ⊕⊕⊕⊕ |
| **Rooks et al.**  **2020 b** | RCT | No concern | No concern | No concern | No concern | No concern | ⊕⊕⊕⊕ |
| **Heymsfield et al. 2021** | RCT | No concern | No concern | No concern | No concern | No concern | ⊕⊕⊕⊕ |

Reference: Ryan R, Hill S (2016) How to GRADE the quality of the evidence. Cochrane Consumers and Communication Group, available at http://cccrg.cochrane.org/author-resources. Version 3.0 December 2016.

Link: <https://neonatal.cochrane.org/sites/neonatal.cochrane.org/files/uploads/how_to_grade.pdf>
