# Supplementary material for: Effect of Bimagrumab on body composition: a systematic review and meta-analysis
Source: Aging Clin Exp Res. 2024 Sep 9;36(1):185. doi: 10.1007/s40520-024-02825-4 (PMC11385021; doi:10.1007/s40520-024-02825-4)
Supplement: Supplementary file 3 — Supplementary file3 (DOCX 16 KB) [file 40520_2024_2825_MOESM3_ESM.docx]

Supplementary Table X. Leave-one-out meta-analysis approach for the three outcomes.

| **Thigh Muscle Volume, %** | | | | | |
| --- | --- | --- | --- | --- | --- |
| *Study* | *MD* | *95%CI* | *P (for study effects)* | *I^2^, %* | *P (for heterogeneity)* |
| Rooks 2017a | 5.24 | 3.82 to 6.67 | <0.001 | 53 | 0.09 |
| Rooks 2017b | 5.20 | 3.90 to 6.51 | <0.001 | 52 | 0.10 |
| Polkey 2018 | 4.76 | 3.28 to 6.25 | <0.001 | 40 | 0.17 |
| Rooks 2020a | 5.87 | 4.50 to 7.25 | <0.001 | 0 | 0.85 |
| **Lean Body Mass, Kg** | | | | | |
| *Study* | *MD* | *95%CI* | *P (for study effects)* | *I^2^, %* | *P (for heterogeneity)* |
| Rooks 2020a | 1.90 | 1.57 to 2.23 | <0.001 | 52 | 0.08 |
| Rooks 2020b | 1.85 | 1.37 to 2.33 | <0.001 | 41 | 0.13 |
| Heymsfield 2021 | 1.84 | 1.48 to 2.21 | <0.001 | 39 | 0.15 |
| Hofbauer 2021 | 1.98 | 1.59 to 2.38 | <0.001 | 0 | 0.92 |
| **Fat Body Mass, Kg** | | | | | |
| *Study* | *MD* | *95%CI* | *P (for study effects)* | *I^2^, %* | *P (for heterogeneity)* |
| Rooks 2020a | -4.56 | -5.09 to -4.03 | <0.001 | 96 | <0.001 |
| Rooks 2020b | -7.18 | -8.34 to -6.02 | <0.001 | 11 | 0.33 |
| Heymsfield 2021 | -3.82 | -4.42 to -3.23 | <0.001 | 0 | 0.81 |
